# Public Analysts

**Published:** 1878-06

**Authors:** 


Public Analysts.—The *Societe d'Hygiene*, of Paris, is making arrangements to establish in the cities and towns of France chemical laboratories for the purpose of examining articles of food and detecting adulterations or unhealthful constituents.

